# Tea and Coffee

**Published:** 1869-11

**Authors:** 


### TEA AND COFFEE.

TAKING into account the habits of the people, tea and coffee, for supper and breakfast, add to human health and life, if a single cup be taken at either meal, and is never increased in strength, frequency, or quantity. If they were mere stimulants, and were taken thus in moderation and with uniformity, they would, in time, become either inert, or the system would become so habituated to their employment, as to remain in the same relative position to them, as if they had never been used; and, consequently, as to themselves, they had better never have been used, as they are so liable to abuse. But science and fact unite in declaring them to be nutritious, as well as stimulant; hence, they will do a new good to the system every day, to the end of life, just as bread and fruits do: hence, we never get tired of either. But the use of bread and fruits is daily abused by multitudes, and dyspepsia and cholera morbus result; yet, we ought not to forego their employment on that account, nor should we forego the use of tea and coffee because their inordinate use gives neuralgies and other ailments.

But the habitual use of tea and coffee, at the last and first meals of the day, has another high advantage, is productive of incalculable good in the way of averting evils.

We will drink at our meals, and if we do not drink these, we will drink what is worse—cold water, milk, or alcoholic mixtures. The regular use of these last will lead the young to drunkenness; the considerable employment of simple milk, at meals, by sedentary people—by all, except the robust—will either constipate, or render bilious; while cold water largely used, that is to the extent of a glass or two at a meal, especially in cold weather, attracts to itself so much of the heat of the system, in raising said water to the temperature of the body—about one hundred degrees—that the process of digestion is arrested; in the meanwhile, giving rise to a deathly sickness of stomach, to twisting pains, to vomitings, purgings, and even to cramps, to fearful contortions, and sudden death; which things would have been averted, had even the same amount of liquid, in the shape of simple hot water, been used. But any one knowing these things, and being prejudiced against the use of tea and coffee, would subject himself to be most unpleasantly stared at, and questioned, if not ridiculed, were he to ask for a cup or glass of hot water. But, as tea and coffee are now universal beverages, are on every table, and every body is expected to take one or the other as a matter of course, they are unwittingly the means of safety and of life to multitudes. They save life, where a glass of cold water would have destroyed it. So that the use of these beverages is not merely allowable, it is politic, it is a necessity.

